# Oxytocin receptor gene polymorphism (rs53576) and digit ratio associates with aggression: comparison in seven ethnic groups

**DOI:** 10.1186/s40101-020-00232-y

**Published:** 2020-08-14

**Authors:** Marina Butovskaya, Victoria Rostovtseva, Polina Butovskaya, Valentina Burkova, Daria Dronova, Vasilisa Filatova, Eugenia Sukhodolskaya, Vasiliy Vasiliev, Tania Mesa, Araceli Rosa, Oleg Lazebny

**Affiliations:** 1 Institute of Ethnology and Anthropology, Russian Academy of Sciences, Leninsky Prospect 32a, 119991 Moscow, Russia; 2 National Research University Higher School of Economics, Moscow, Russia; 3 Vavilov Institute of General Genetics RAS, Moscow, Russia; 4 Federal Budget Institution of Science “Central Research Institute of Epidemiology” of The Federal Service on Customers’ Rights Protection and Human Well-being Surveillance, Moscow, Russia; 5 Institute of Gene Biology, Russian Academy of Sciences, Moscow, Russia; 6 Secció de Zoologia i Antropologia Biològica, Departament de Biologia Evolutiva, Ecologia i Ciències Ambientals, Facultat de Biologia, Universitat de Barcelona (UB), Barcelona, Spain; 7 Institut de Biomedicina de la Universitat de Barcelona (IBUB), Barcelona, Spain; 8 Centre for Biomedical Research Network on Mental Health (CIBERSAM), Instituto de Salud Carlos III, Barcelona, Spain; 9 Koltzov Institute of Developmental Biology, Russian Academy of Sciences, Moscow, Russia

**Keywords:** *OXTR* rs53576, Digit ratio, 2D:4D, Buss-Perry Aggression Questionnaire, Ethnic groups, Men, Women, Europeans, Asians, Africans

## Abstract

**Background:**

The specific role of the oxytocin receptor (*OXTR*) gene polymorphisms in emotional support seeking, related to social norms and culturally normative behavior, has been discussed in several studies. Evidence on the association between aggression and *OXTR* polymorphisms has also been reported. The goal of the current study was to analyze the effect of the *OXTR* rs53576 polymorphism, prenatal testosterone effect (second-to-fourth digit ratio, or 2D:4D), and culture on aggression assessed with the Buss-Perry Aggression Questionnaire (BPAQ).

**Methods:**

The data were collected in Russia and Tanzania and included seven ethnic groups of European, Asian, and African origin. The total sample included 1705 adults (837 males, 868 females). All the subjects were evaluated with the BPAQ. As a measure of prenatal androgenization, the second and fourth digits were measured directly from hand, and the digit ratios were calculated. All the participants provided buccal samples, from which genomic DNA was extracted, and the *OXTR* gene rs53576 polymorphism was genotyped. Statistical analysis was performed using SPSS version 23.0; the alpha level for all analyses was set at 0.05.

**Results:**

The ethnic group factor was the most significant predictor of ratings on BPAQ (medium effect size for physical aggression, anger and hostility scales, and low for verbal aggression). To study the effect of sex, the *OXTR* polymorphism, and prenatal androgenization, we conducted the *z*-score transformation for BPAQ scales and 2D:4D for each ethnic group and pooled these data into new *z*-score variables. According to the GLM analysis after leveling the effects of culture (z-transformation), all four scales of BPAQ demonstrated association with sex (main effects), with men scoring higher on physical and verbal aggression and women scoring higher on anger and hostility. Anger and hostility scales were also associated with *OXTR* polymorphism and 2D:4D of the right hand. The lowest levels of anger and hostility were observed in individuals with the AA genotype, especially in men.

**Conclusions:**

Our data suggest that both oxytocin (*OXTR* gene polymorphism) and fetal testosterone (2D:4D) may significantly affect emotional (anger) and cognitive (hostility) aggression in humans, given the leveling the role of culture.

## Background

As the capacity to form social bonds and parental behavior, general sociality is a cornerstone of human society, deeply rooted in human evolution [1, 2]. The way of how prosocial behavior develops through interactions between culturally varying norms, social cognition, emotions, and, potentially, genes, is at the center of attention for numerous theoretical and empirical studies [3]. Through social norms, humans are intrinsically motivated to enforce rules of social co-existence and cooperation, as well as rules of punishment in the direction of those who do not behave prosocially [4], and do not punish violators [5]. Strong tendency to follow and enforce social norms in our species turns these norms into a powerful tool for enforcing cooperation at a large scale [6]. “Culturally constructed environments create powerful – and often autocatalytic – selection pressures on genes” [7]. Currently accumulated data suggest that human culture has impacted the modern human genome and its variation [8, 9].

It may be useful to keep in mind this perspective while discussing the gene-environmental interactions in the expression of human aggression. Human aggression is multi-dimensional and may be expressed physically, verbally, emotionally (e.g., anger), or cognitively (e.g., impulsivity, hostility) [10]. There are multiple risk factors that directly or indirectly influence aggression, including age, sex, genetics, and psychopathological and environmental factors [11, 12]. Men are exhibiting more violent (physical) same-sex aggression than women in most cultures [13–17], which may be attributed to the higher impulsiveness of men and a stronger fear of physical danger in women. On the other hand, the critical finding in this respect is that cultural and religious norms affect the personal potential for aggressive behavior, regulating physical or verbal expression, reducing or reinforcing their level, and regulating emotional and cognitive potential.

The role of oxytocin in human social integration, as well as aggression, deviant, and antisocial behavior, has been at the focus of attention during recent years. Oxytocin neuropeptide, produced by the hypothalamus and secreted by the pituitary gland, has been frequently mentioned as an essential regulator of mother-child attachment, social affiliation, and social bonding [18, 19]. Oxytocin’s secretion decreases anxiety and protects against stress [18], increases empathy and trust [20, 21], and positively affects generosity in the context of perspective-taking [22]. Some authors also report on its role in group-serving dishonesty/deception, by promoting dishonesty when the outcome favored the group to which an individual belonged [23]. Oxytocin produces its effects through oxytocin receptor (OXTR) [24], which is present both in the brain and other body tissues and is involved in the development of the social brain [25]. Since the discovery of the *OXTR* gene structure [26], numerous studies have focused on the association between *OXTR* gene polymorphisms and different aspects of human physiology and behavior. The genetic variation of the *OXTR* affects the influence of deviant peer affiliation on antisocial behavior and is associated with proactive aggression, but not with reactive aggression [27]. Variation in the *OXTR* gene is commonly assessed through single nucleotide polymorphisms (SNPs). The interaction effect of the two *OXTR* SNPs, rs1488467 and rs4564970, with alcohol consumption on trait anger was reported for the Finnish sample of men and women [28]. The A allele at rs53576 of the *OXTR* gene was associated with prior suicide attempts, and neither abuse history nor attachment style moderated this relationship [29]. Data on Chinese Han adolescent highlight the compound effect of stressful life events on aggression and provided evidence of the relationship between the oxytocin system and aggressive behavior [30]. High life stress during the past 12 months was associated with high levels of physical aggression and hostility in *OXTR* rs53576 AA carriers, but not in G carrier boys, but this association was not significant for girls. The relevance of *OXTR* rs237885 to aggression was also demonstrated. Mainly, rs237885 TT carriers with a history of childhood abuse had a higher risk of aggression [31]. The specific role of *OXTR* gene polymorphisms in emotional support seeking, related to social norms and culturally normative behavior, has been discussed in several studies [32–37], with *OXTR* rs53576 being one of the main focuses of attention. *OXTR* rs53576 has been related to attachment security and marital satisfaction, and GG carriers rated higher compared A carriers [38]. Li and colleagues reported a positive association between the rs53576 polymorphism and general sociality and concluded that the G allele homozygotes had higher general sociality than the A allele carriers [39]. Kim and colleagues [32] found that emotional support seeking was more evident in distressed North Americans having the G allele at the *OXTR* rs53576 than in those with the AA genotype, but not in Koreans, whose emotional support seeking is not a culturally normative behavior. Later, the same authors reported that Koreans with the GG genotype were more likely to use emotional suppression than those with the AA genotype and hesitated to seek emotional support in response to stress, which is more in line with normative behavior in Korea [32]. Individuals carrying the G allele of the *OXTR* rs53576 appeared to be more sensitive to the cultural environment [37]. The general conclusion might be that certain people regulate emotions according to cultural norms because they are biologically susceptible to be sensitive to the socio-emotional cues in a culture [36]. However, the only previous cross-sectional study, analyzing the association between rs53576 and a wide variety of emotional traits and states in a sample of young adults of various ethnicities (European, Asian, Maori/Pacific Islander, others), did not find any significant associations [40].

Another focus of the current study is the impact of prenatal androgen exposure on the aggressive behavior and its possible association with *OXTR* effects. Digit ratio (2D:4D), as a possible proxy to fetal androgenization, has been discussed in numerous studies [41–44]. Sexual dimorphism of the digit ratios is found across the majority of species starting from amphibians [45, 46], although it is not unidirectional in all species [47, 48]. In humans, 2D:4D is also known to be sexually dimorphic, with males having lower ratios than females in the majority of studied populations [15, 16, 49–51] (although see [52–54] for criticism). For today, the direct evidence on the association between digit ratios and prenatal androgen concentrations in humans is still quite scarce and somewhat contradictive [55–57]. However, a part of the mechanisms underlying the impact of prenatal androgen/estrogen exposure on the formation of differences in the 2nd and 4th finger lengths has been revealed in mice [58]. The genetic basis linking gonads’ and digits’ development has also been investigated during recent years [59–61]. It is well-known that in men, testosterone concentrations have two peaks during the lifespan: (1) in the period of early prenatal development (peak at ~ 24th week of gestation) [62] and (2) in puberty. The fetal and postnatal Leydig cells, which are responsible for testosterone production during prenatal and pubertal periods respectively, most likely represent morphologically and functionally different cell generations with different origins [63, 64]. This is also supported by a well-known lack of association between the 2D:4D ratio and adult testosterone levels in humans [65–67]. The latter suggests an existence of a relatively independent path for basic masculinization through exposure to androgens in utero. The period of the prenatal testosterone peak takes place at the time of the early brain maturation, which may play an important role in the formation of the sexual dimorphism of human brain structures [68–71] and concomitant behavior. Of particular interest, within the scope of the current study, are findings on associations between the digit ratio and personality traits [72], including aggression at various stages of life history [73–77], risk-taking [78–81], higher physical aggression in adult men, but not women [73, 82], as well as adolescent boys but not girls [15, 17]. Data from various studies pointed on the mutual influence of oxytocin and testosterone systems on the development of social cognition in humans [83]. In experiments with oxytocin administration versus placebo, conducted on men, individuals with a higher digit ratio (low fetal testosterone) preferred to include low-threat, rather than high-threat targets, into their groups [84]. On the contrary, men with high testosterone exposure in the uterus, after intranasal oxytocin treatment, preferred to select high-threat targets more than controls. According to other studies [85], the administration of oxytocin increased plasma testosterone in young men. Many studies currently are taking efforts to reveal the magnitude of environmental and genetic effects on aggression [86–93], as well as antisocial behavior [94].

It seems clear that genes may shape psychological predispositions, but also that culture might influence how these predispositions are behaviorally manifested, and culture provides particular contexts that afford opportunities and constraints for the development of psychological tendencies by presenting cultural, social norms, and limitations [34, 75, 89, 95]. Given that genes and culture may interact to produce different outcomes, we tested the three-way interaction of genes, culture, and sex in predicting direct aggression, such as physical and verbal aggression; aggressive emotional feelings, such as anger; and cognitive component, such as hostility. To this aim, we investigated the association between culture (ethnic group, religion), sex, *OXTR* gene SNP rs53576, digit ratio, and self-ratings on aggression assessed with the Buss-Perry Aggression Questionnaire (BPAQ) [10].

The following hypotheses were tested: (1) self-ratings on aggression in men and women are significantly influenced by cultural norms and prescriptions (socialization); (2) the *OXTR* rs53576 polymorphism may cause differences in BPAQ self-ratings, especially in emotional dimension of aggressive manifestations; (3) the *OXTR* rs53576 effect on BPAQ ratings is sex-specific; and (4) the digit ratio associations with self-ratings on BPAQ may be different in A-carriers, compared to GG *OXTR* rs53576 genotypes.

## Methods

### Participants and procedure

The sample included 1705 adults from seven ethnic groups of European, Asian, and African origin. Four ethnic groups were from Russia (Russians [89], Tatars [75], Ob-Ugric [96, 97], and Buryats [75, 98]) with the mean age of 21 years. These samples were collected between 2010 and 2019. Three ethnic groups were sampled from Tanzania between 2006 and 2014 (Hadza [16, 74, 87–89, 97, 99–102], Datoga [16, 88, 89, 97, 99–103], and Isanzu), with a mean age of 36 years. These groups are representatives of traditional African cultures. More information about these cultures may be found elsewhere [15, 16, 74, 87–89, 96–105].

All the Russian participants gave written informed consents, while in Tanzania, as most of the participants were illiterate there, the oral informed consents were provided, following the Declaration of Helsinki. The study was approved by the Institute of Ethnology and Anthropology of the Russian Academy of Sciences (RAS) and the Commission for Science and Technology of Tanzania (COSTECH).

### Laboratory methods

All the participants provided buccal samples. Genomic DNA was isolated using Diatom DNA Prep 200 (Isogen Lab, Moscow, Russia). DNA quality from all the samples was assessed by spectrophotometer readings (A260/280) using NanoDrop.

The polymorphism of the *OXTR* gene (rs53576) was genotyped using Taqman 5′ exonuclease assay (Applied Biosystems). The probes for genotyping were ordered through the TaqMan SNP genotyping assays (ID: C___3290335_10) Applied Biosystems assay-on-demand service. The final volume was 5 μl, which contained 5 ng of genomic DNA, 2.5 μl of Taqman Master Mix, and 0.25 μl of 40 genotyping assays. Polymerase chain reaction plates were read on an ABI PRISM 7900HT instrument, and SDS v2.3 software (Applied Biosystems) was used for the genotype analysis of data.

All population statistical data processing was carried out using GenAlEx software v6.5 [106, 107]: genotype and allele frequencies, Hardy-Weinberg equilibrium (HWE) test, test of homogeneity, linkage disequilibrium test, estimations of heterozygosity, and fixation index (FST) and their significances.

### Assessment of aggression

Self-reported aggression was assessed with the Buss-Perry Aggression Questionnaire (BPAQ) [10]. The BPAQ includes 29 statements, grouped into four scales—physical aggression (9 items), verbal aggression (5 items), anger (7 items), and hostility (8 items)—answered on a Likert scale anchored by 1 (extremely uncharacteristic of me) and 5 (extremely characteristic of me). The translation of the BPAQ into Russian and Swahili was done, following accepted standards (translations and back translations by four bilingual assistants [108, 109]), and translated versions have been already used in several previous studies [15–17, 74, 75, 78, 87–89, 96].

In Russia, all the participants filled out the forms by themselves. In Tanzania, almost all respondents were illiterate; thus, they were personally interviewed in Swahili by the first author or a trained local assistant. Consequently, for Tanzanian respondents, all questions were read aloud in one-to-one dialogs, and further explanations were provided, if necessary. In Tanzania, the local assistant provided examples of actions related to the trait common in the culture to ensure that participants understood the questions. Scores on individual scales and total BPAQ scores were calculated only for respondents who answered all items. Cronbach’s alpha for the total scores was 0.77 for the Russian sample, and 0.79 for the Tanzanian, and ranged between 0.60 and 0.70 for separate scales in both samples.

### Anthropometrical assessment

The second and fourth digits of the participants were measured twice with a Vernier caliper, measuring to 0.01 mm, from the basal crease to the tip of the finger. Mean values of the two measurements were used to calculate the 2D:4D ratios. In this study, we provide the data on the right-hand digit ratio (R2D:4D), given the general information that the right-hand digit ratio usually is more sexually dimorphic compared to the left hand [110]. In the cases where there was a band of creases at the base of the digit, the most proximal crease was used [111]. Participants who reported injuries or deformities of the second or fourth digits were excluded from the statistical analysis. All measurements were done by experienced anthropologists.

The 2D:4D ratios were calculated following the procedure described earlier [112]. The repeated measures of the first and second digits for the whole sample gave an intra-class correlation of 0.94. Hence, it was assumed that the differences in between-individual measurements of the 2D:4D were significantly higher than the within-individual measurement error.

### Statistical analyses

Mean and standard deviation (SD) for continuous variables was used to describe the sample’s characteristics. *T* test was used to estimate the sex differences in ratings on BPAQ scales in each of the studied groups, as well as for estimating differences in aggression between AA and G-allele carriers. The main and interaction effects of ethnic group, sex, and *OXTR* genotypes on four scales of the BPAQ were calculated using multivariate analysis of variance (MANOVA). Due to the contradictory results obtained in MAN(C)OVA, the effect of the *OXTR* gene rs53576 on the z-transformed self-ratings on the BPAQ scales was tested with one-way analysis of variance (ANOVA), followed by the use of the Dunnett’s T3 post hoc test [113, 114]. This approach allowed to solve the problems related to unequal sample sizes and unequal SDs and to test for differences among *OXTR* genotype groups separately in men and women. One-way ANOVA was used for analysis of the z-transformed scales of BPAQ for male and female samples to estimate the effect of *OXTR* genotypes. Univariate analysis of covariance (ANCOVA) was used to assess the impact of sex, digit ratio, and *OXTR* rs53576 on the BPAQ aggression scales after leveling the cultural effects. Linear regression was used to test the effects of the right-hand 2D:4D and rs53576 polymorphism on the BPAQ scales. Statistical analysis was performed using SPSS version 23.0 (IBM Corp., Armonk, NY, USA). The alpha level for all analyses was set at 0.05.

## Results

### Genotype distribution

**Fig. 1:**
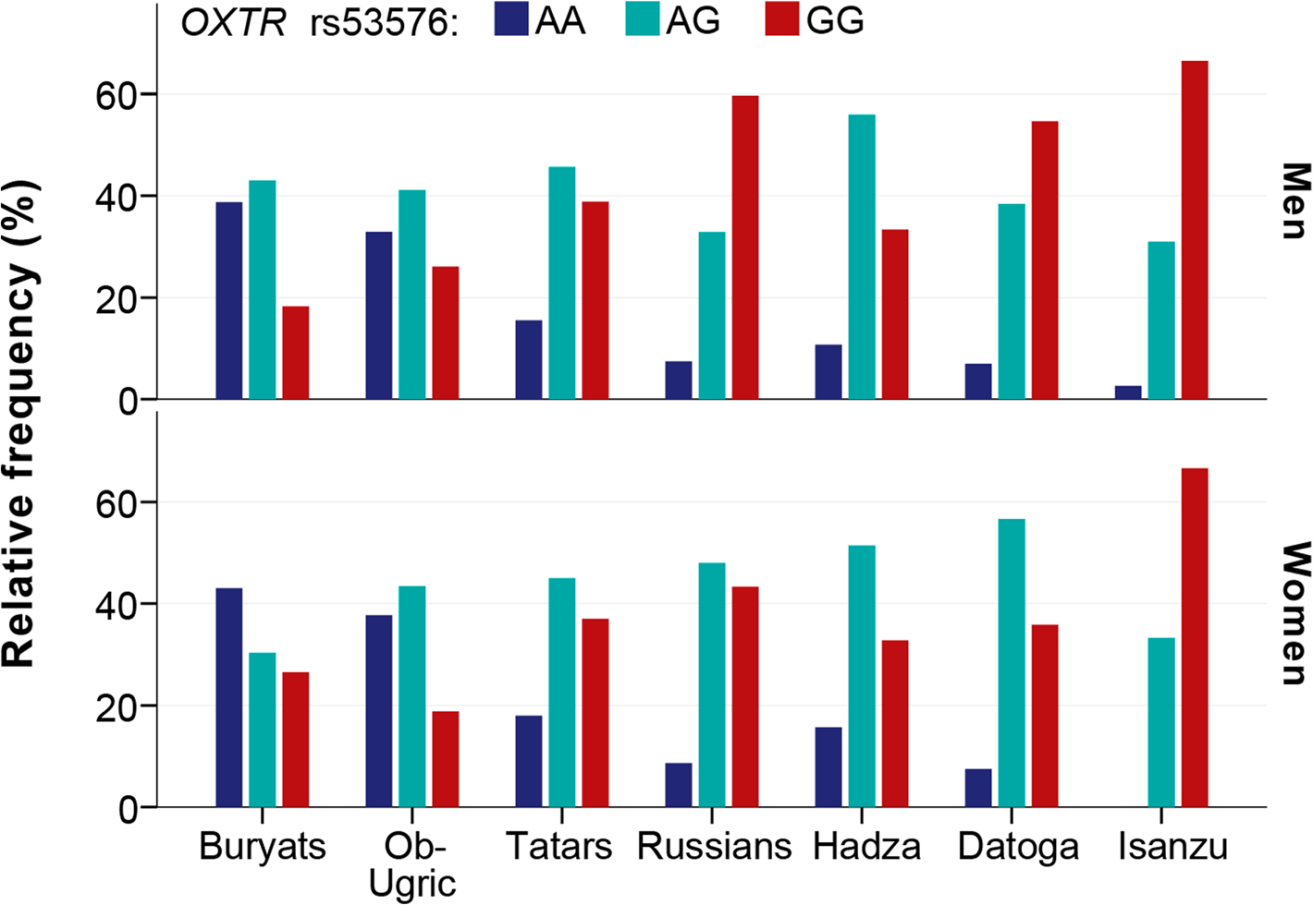
Distributions of the *OXTR* rs53576 genotypes by sex in the seven studied populations. The steady increase in proportion of GG genotypes in tested populations from east (Asia) to west (Europe and Africa)

The *OXTR* rs53576 genotype and allele frequencies are presented in Table 1. The genotype distributions by sex are presented in Fig. 1. As seen from Table 1, the frequencies of the alleles A and G are gradually changing from east of Asia (Buryats) to Europe (Russians and Tatars) and Africa. In the Asian populations, allele A prevails over allele G and vice versa in the rest of populations. Accordingly, genotypes AA and AG are prevailing in Buryats and Ob-Urgic people, while AG and GG genotypes in Russians, Tatars, and African populations. As *χ*^2^ test was applied for HWE several times, the Benjamini-Hochberg approach was used, and the corrected level of significance (*p* < 0.05) was obtained, *q* = 0.007. Examining the individual *p* values justified HWE for all samples, including the Buryat sample, accepting its null hypothesis rejection as accidental.

**Table 1:** Genotype and allele frequencies of the *OXTR* rs53576 and HWE in the samples studied

Population	***N***	Frequency					HWE	
		Genotype			Allele			
		AA	AG	GG	A	G	*χ*^2^	*P*
Buryats	172	0.407	0.372	0.221	0.593	0.407	9.030	0.003
Ob Ugric	248	0.363	0.427	0.210	0.577	0.423	3.851	0.05
Tatars	203	0.167	0.453	0.379	0.394	0.606	0.528	0.467
Russians	218	0.083	0.431	0.486	0.298	0.702	0.226	0.634
Hadza	317	0.129	0.539	0.331	0.399	0.601	4.930	0.05
Datoga	345	0.072	0.475	0.452	0.310	0.690	4.243	0.05
Isanzu	202	0.020	0.317	0.663	0.178	0.822	1.347	0.246

*HWE* Hardy-Weinberg equilibrium

### Ethnic group, sex, OXTR genotype, and 2D:4D as predictors of aggression

**Fig. 2:**
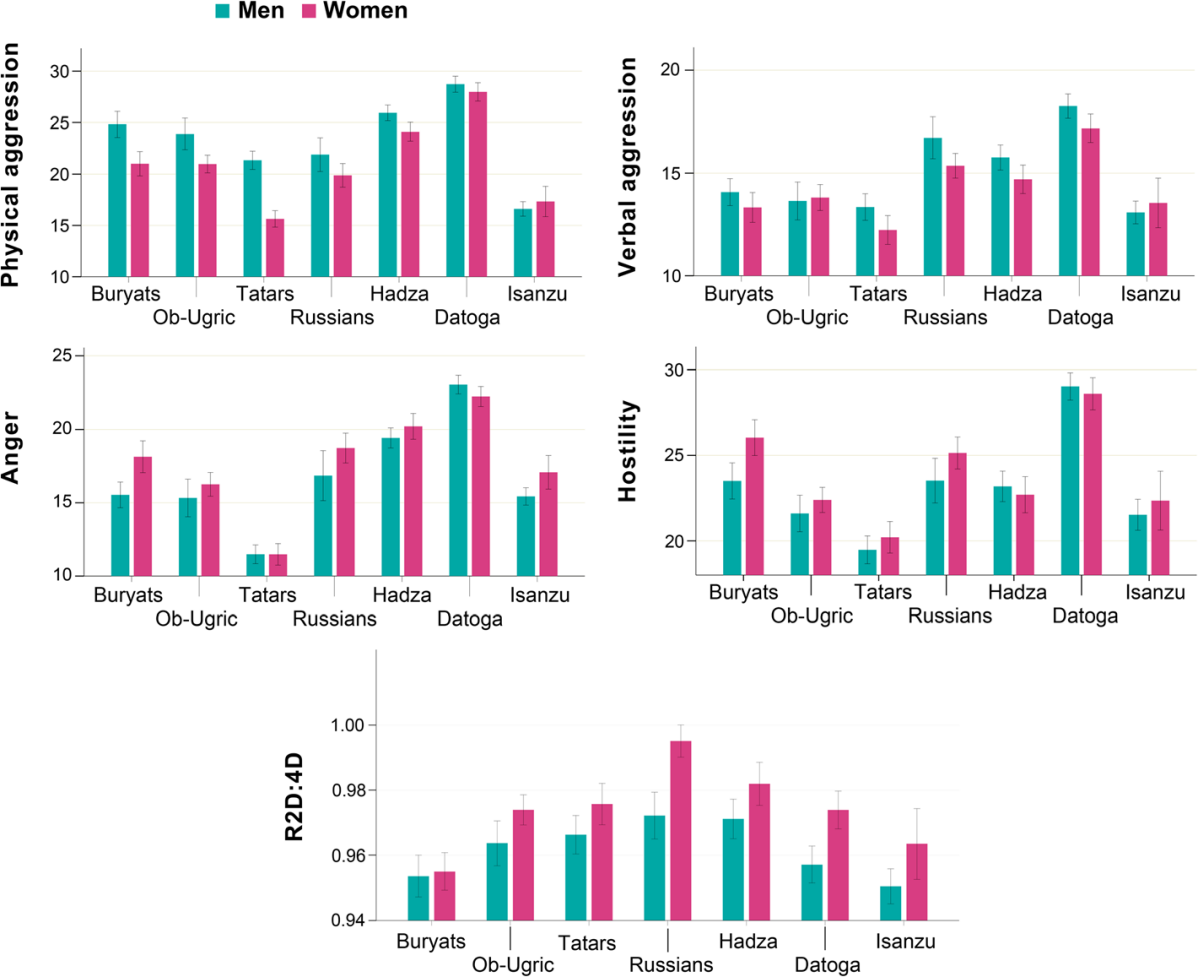
Distributions of BPAQ scales and right-hand 2D:4D for men and women from seven studied populations. R2D:4D—digit ratio of the right hand. Mean scores for BPAQ scales are presented as mean total scores per each scale. Inter-scale comparisons of raw total scores are not assumed, since each scale contains different number of questions

Mean scores for physical aggression (PA), verbal aggression (VA), anger (AN), and hostility (HT), assessed with the BPAQ and the 2D:4D ratio in the seven ethnic groups and by sex, are shown in Table 2. Sexual differences were found almost in all four aggression scales and for the 2D:4D ratio (Fig. 2).

**Table 2:** Descriptive statistics and sex differences in aggression scales (BPAQ) and right-hand 2D:4D ratio

BPAQ scale	Population	***N***	Men		Women		***t***	***p*** (sig.)
			Mean	SD	Mean	SD		
PA	Buryats	172	24.8	6.1	21.0	5.3	4.339	< 0.001
	Ob-Ugric	248	23.9	6.5	21.0	5.8	3.494	0.001
	Tatars	203	21.3	4.5	15.6	4.1	9.440	< 0.001
	Russians	217	21.9	6.7	19.9	7.1	1.957	0.052
	Hadza	317	25.9	5.1	24.1	5.4	3.048	0.002
	Datoga	345	28.7	5.2	28.0	6.0	1.246	0.214
	Isanzu	203	16.6	4.4	17.3	5.3	− 0.962	0.337
VA	Buryats	172	14.1	3.2	13.3	3.2	1.517	0.131
	Ob-Ugric	248	13.6	4.0	13.8	4.2	− 0.290	0.772
	Tatars	203	13.3	3.3	12.2	3.5	2.328	0.021
	Russians	217	16.7	4.2	15.4	3.7	2.393	0.018
	Hadza	317	15.8	4.1	14.7	4.2	2.272	0.024
	Datoga	345	18.3	3.9	17.2	4.6	2.333	0.020
	Isanzu	203	13.1	3.5	13.5	4.3	− 0.778	0.438
AN	Buryats	172	15.5	4.3	18.1	4.8	− 3.749	< 0.001
	Ob-Ugric	248	15.3	5.5	16.3	5.4	− 1.223	0.223
	Tatars	203	11.5	3.3	11.5	3.7	0.011	0.991
	Russians	217	16.9	7.0	18.7	6.3	− 1.964	0.051
	Hadza	317	19.4	4.6	20.2	5.3	− 1.424	0.155
	Datoga	345	23.1	4.2	22.3	4.6	1.721	0.086
	Isanzu	203	15.4	3.7	17.1	4.1	− 2.675	0.008
HT	Buryats	172	23.5	5.1	26.0	4.7	− 3.362	0.001
	Ob-Ugric	248	21.6	4.6	22.4	4.9	− 1.176	0.241
	Tatars	203	19.5	4.2	20.2	4.6	− 1.168	0.237
	Russians	217	23.5	5.3	25.1	5.8	− 1.945	0.053
	Hadza	317	23.2	6.0	22.7	6.3	0.699	0.485
	Datoga	345	29.0	5.3	28.6	6.3	0.686	0.493
	Isanzu	203	21.5	5.7	22.4	6.1	− 0.884	0.378
R2D:4D	Buryats	172	0.95	0.03	0.96	0.03	− 0.329	0.743
	Ob-Ugric	248	0.96	0.03	0.97	0.03	− 2.397	0.017
	Tatars	203	0.97	0.03	0.98	0.03	− 2.152	0.033
	Russians	217	0.97	0.03	1.00	0.03	− 5.132	< 0.001
	Hadza	317	0.97	0.04	0.98	0.04	− 2.365	0.019
	Datoga	345	0.96	0.04	0.97	0.04	− 4.075	< 0.001
	Isanzu	203	0.95	0.03	0.96	0.04	− 2.297	0.023

Sex differences presented according to Student’s *T* test (*t*—test statistics, *p* – statistical significance)

*PA* Physical aggression, *VA* Verbal aggression, *AN* Anger, *HT* Hostility, *R2D:4D* Digit ratio of the right hand

The MANOVA two-way analysis with ratings on four aggression scales as dependent variables; the ethnic group, sex, and rs53576 *OXTR* polymorphism as independent variables; and the two-way interaction effects of independent variables was conducted (Table 3).

**Table 3:** The impact of sex, ethnicity, and rs53576 *OXTR* polymorphism on individual aggression ratings

Predictor	Dependent variable	***F*** (df)	***p*** (sig.)	Partial eta^**2**^
Sex	Physical aggression	51.78 (1)	< 0.001	0.030
	Verbal aggression	7.73 (1)	0.005	0.005
	Anger	14.49 (1)	< 0.001	0.009
	Hostility	7.94 (1)	0.005	0.005
Ethnic group	Physical aggression	53.48 (6)	< 0.001	0.161
	Verbal aggression	26.28 (6)	< 0.001	0.086
	Anger	80.83 (6)	< 0.001	0.225
	Hostility	42.48 (6)	< 0.001	0.132
*OXTR* rs53576	Physical aggression	1.43 (2)	0.240	0.002
	Verbal aggression	1.44 (2)	0.236	0.002
	Anger	0.92 (2)	0.399	0.001
	Hostility	1.77 (2)	0.171	0.002
Sex × ethnic group	Physical aggression	6.12 (6)	< 0.001	0.021
	Verbal aggression	1.61 (6)	0.140	0.006
	Anger	3.89 (6)	0.001	0.014
	Hostility	2.45 (6)	0.023	0.009
*OXTR* rs53576 × sex	Physical aggression	0.43 (2)	0.649	0.001
	Verbal aggression	0.22 (2)	0.800	< 0.001
	Anger	1.43 (2)	0.240	0.002
	Hostility	3.06 (2)	0.047	0.004
*OXTR* rs53576 × ethnic group	Physical aggression	0.90 (12)	0.547	0.006
	Verbal aggression	0.92 (12)	0.521	0.007
	Anger	2.50 (12)	0.003	0.018
	Hostility	0.93 (12)	0.519	0.007

MANOVA two-way analysis with the BPAQ scales ratings as dependent variables; ethnic group, sex, and *OXTR* gene polymorphism (rs53576) as independent factors; and the two-way interaction effects of these factors are presented

*F* test statistics, *df* Degrees of freedom, *p* statistical significance, *partial eta*^*2*^ effect size

These three independent factors, along with their two-way interactions, explained between 35.4 and 18.1% of variations of scores on four BPAQ scales. The ethnic group factor was the most significant predictor (the medium effect sizes for physical aggression, anger and hostility scales, and low for verbal aggression). Post hoc Bonferroni tests revealed highly significant differences between seven tested groups on physical aggression, anger, and hostility (*p* ranges from 4.217E−25 to 0.001).

The main effect of sex was significant for all scales, however, with small effect sizes. The effect of *OXTR* rs53576 polymorphism on aggression was not significant under such conditions. However, we found that the interaction effect of ethnic group × *OXTR* was significant for the anger scale, and the *OXTR* × sex interaction for the hostility scale. In both of these cases, the effect sizes were small (Table 3). The interaction effect of ethnic group × sex was significant for all scales but verbal aggression (again, the effect sizes were small) (Table 3).

Given the prevailing impacts of the ethnic origin and sex on the studied aggression parameters, possible effects of the *OXTR* gene polymorphism on aggression could appear to be masked by such a strong population-specific and sex-specific effects. Hence, to reveal the possible impact of the *OXTR* on aggression independently of culture and sex, we have leveled the aggression measures for all studied ethnic groups and analyzed males and females separately. Leveling the population effects was achieved through the procedure of z-transformation of the BPAQ scales for each of the ethnic groups with subsequent pooling of these data into new z-transformed variables (z-BPAQ scales). They were later tested separately for males and females using one-way ANOVA with *OXTR* as an independent factor. In men, the ANOVA revealed a significant effect of *OXTR* genotypes on BPAQ anger (*F* = 3.06, df1 = 2, df2 = 834, *p* = 0.047) and hostility (*F* = 3.68, df1 = 2, df2 = 834, *p* = 0.026) scales. The post hoc Dunnett’sT3 test (for unequal variances) has revealed that the contrasting genotypes were AA and GG (*p* = 0.031 and *p* = 0.016, accordingly). The effects were small, but significant (anger: *d* = 0.26 and *ɳ*^2^ = 0.007; hostility: *d* = 0.28 and *ɳ*^2^ = 0.009).

**Fig. 3:**
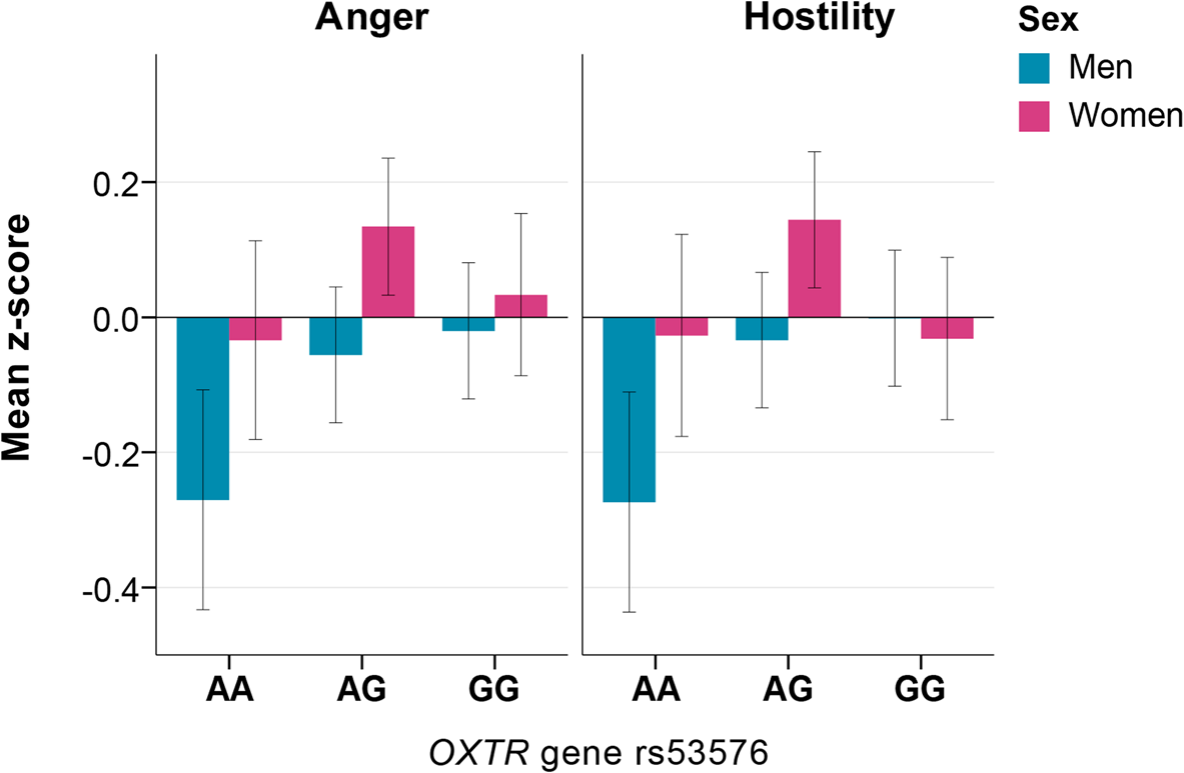
Differences in anger and hostility (*z*-scores) between carriers of different *OTXR* rs53576 genotypes. Significant differences in anger (*p* = 0.016) and hostility (*p* = 0.018) (*z*-scores) are revealed by the Student’s *T* test between carriers of AA genotype of *OXTR* gene (SNP rs53576) and others (G allele carriers) in general sample (*N* = 1705). These differences were especially pronounced in men (*N* = 837), whereas within women (*N* = 868), differences were not statistically significant. Anger. Men: one-way ANOVA (*F* = 3.06, df = 2, df = 834, *p* = 0.047), Dunnett T3 test (AA vs. GG, *p* = 0.031). Women: not significant. Hostility. Men: one-way ANOVA (*F* = 3.68, df1 = 2, df2 = 834, *p* = 0.026), Dunnett T3 test (AA vs. GG, *p* = 0.016). Women: one-way ANOVA (*F* = 3.096, df = 2, df2 = 865, *p* = 0.046), Dunnett T3 test (not significant)

Among women, the *OXTR* effect was found for hostility scale only (*F* = 3.096, df1 = 2, df2 = 865, *p* = 0.046), but the differences were not significant according to the Dunnett T3 test. To sum up, the lowest levels of anger and hostility were observed in individuals with AA genotype of the *OXTR* rs53576 irrespective of their ethnic identity, and such association was especially pronounced in men (Fig. 3).

**Fig. 4:**
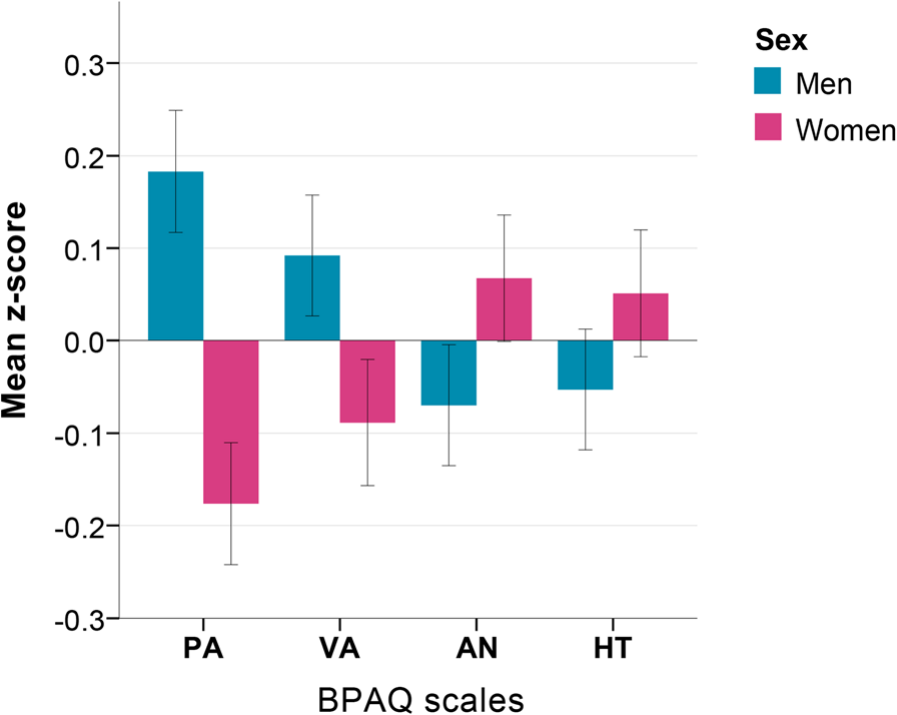
Sex differences in BPAQ subscales (*z*-scores) (at the bottom). BPAQ scales (*z*-scores): PA—physical aggression, VA—verbal aggression, AN—anger, HT—hostility. Sex differences are statistically significant (Student’s *T* test): PA (*p* < 0.001), VA (*p* < 0.001), AN (*p* = 0.004), HT (*p* = 0.032)

Some previous findings suggested the influence of the oxytocin and testosterone systems on shaping human social cognition during early development and mutual contributions of these systems to hypo- or hyper-socio-cognitive manifestations [83]. Consequently, we tested the possible interplay between effects of the *OXTR* polymorphism and the right-hand 2D:4D ratio (as a proxy for prenatal androgenization) after leveling the population differences in aggression ratings and digit ratio (within-population *z*-score standardization) (Table 4). According to the ANCOVA analysis, and corresponding to what has been previously mentioned above (Table 3), all four scales of BPAQ demonstrated association with sex (main effects): men, in comparison with women, generally scored higher on physical and verbal aggression, whereas women scored higher on anger and hostility (sex differences are statistically significant in all cases; Fig. 4).

**Table 4:** Association between aggression subscales (*z*-score), sex, *OXTR* rs53576, and right-hand 2D:4D ratio (*z*-score)

***N***	Trait	Predictors	***F***	***P*** (sig.)	Partial eta^**2**^	Model ***R***^**2**^
1	Physical aggression^Z^	Sex	53.958	**< 0.001**	0.031	0.039
		*OXTR* rs53576	2.401	0.091	0.003	
		R2D:4D^Z^	2.312	0.129	0.001	
		*OXTR* rs53576 * R2D:4D^Z^	0.543	0.581	0.001	
		Sex * *OXTR* rs53576	1.591	0.204	0.002	
		Sex * R2D:4D	1.180	0.277	0.001	
2	Verbal aggression^Z^	Sex	10.991	**0.001**	0.006	0.014
		*OXTR*_rs53576	2.587	0.076	0.003	
		R2D:4D^Z^	0.407	0.524	< 0.001	
		*OXTR* rs53576 * R2D:4D ^Z^	2.011	0.134	0.002	
		Sex * *OXTR* rs53576	0.151	0.859	< 0.001	
		Sex * R2D:4D	0.090	0.764	< 0.001	
3	Anger^Z^	Sex	6.767	**0.009**	0.004	0.018
		*OXTR* rs53576	3.502	**0.030**	0.004	
		R2D:4D^Z^	7.109	**0.008**	0.004	
		*OXTR* rs53576 * R2D:4D^Z^	4.270	**0.014**	0.005	
		Sex * *OXTR* rs53576	0.675	0.509	0.001	
		Sex * R2D:4D	2.470	0.116	0.001	
4	Hostility^Z^	Sex	4.427	**0.036**	0.003	0.015
		*OXTR* rs53576	4.239	**0.015**	0.005	
		R2D:4D^Z^	3.900	**0.048**	0.002	
		*OXTR* rs53576 * R2D:4D^Z^	0.986	0.373	0.001	
		Sex * *OXTR* rs53576	2.228	0.108	0.003	
		Sex * R2D:4D	0.581	0.446	< 0.001	

Four univariate general linear models (ANCOVA) with multiple predictors are presented

*OXTR* (rs53576): AA (*N* = 282), AG (*N* = 755), GG (*N* = 668); sex: male (*N* = 837), female (*N* = 868)

*N* model number; *Trait* dependent variable, one of the four subscales of BPAQ per each model; *Predictors* independent variables, * interactions; *F* test statistics; *P* level of statistical significance (bold: *p* < 0.05); *partial eta*^*2*^ effect size; *Model R*^*2*^ model *R*-squared

^Z^Variable after *z*-score standardization

Physical and verbal aggression, however, were predicted only by sex, with no significant impacts of other predictors (OXTR (rs53576), R2D:4D^Z^), and their interactions. However, the main effect of the R2D:4D ratio on anger was significant only at the level of trend, which occurred due to previously mentioned significant interaction effect between R2D:4D and *OXTR* gene polymorphism (Table 4). To see how these two factors interacted in determining the level of anger, we divided the general sample into three parts according to the type of the *OXTR* rs53576 (AA, AG, GG) and ran linear regression analysis within each type, setting anger (*z*-score) as a response variable, and R2D:4D (z-score) with sex as predictors.

**Fig. 5:**
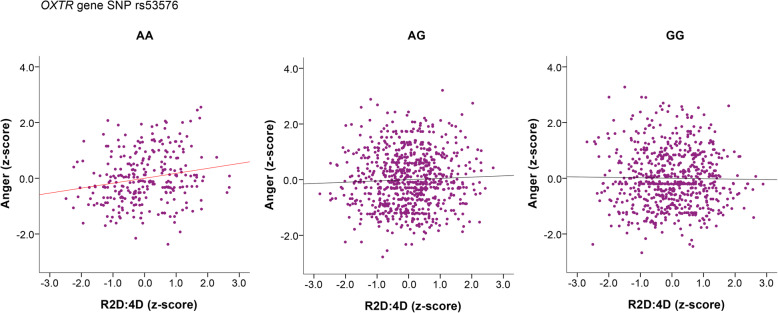
Partial plots for associations between anger and right-hand 2D:4D within individuals distinguished by *OXTR* rs53576. Regression analysis for the association between anger (*z*-score) and 2D:4D ratios of the right hand (R2D:4D; *z*-score) with control for sex: AA carriers (*N* = 282, beta = 0.190, *t* = 3.232, *p* = 0.001; *R*^2^_(model)_ = 0.051, *p*_(model)_ = 0.001); AG carriers (*N* = 755, beta = 0.044, *t* = 1.194, *p* = 0.233; *R*^2^_(model)_ = 0.011, *p*_(model)_ = 0.017); GG carriers (*N* = 668, beta = − 0.016, *t* = − 0.416, *p* = 0.677; *R*^2^_(model)_ = 0.001, *p*_(model)_ = 0.728)

According to the results of the regression analysis, significant association between anger and 2D:4D ratio of the right hand, when controlling for sex, occurred only in carriers of the AA genotype of the *OXTR* gene rs53576 (*N* = 282, beta = 0.190, *t* = 3.232, *p* = 0.001; model *R*^2^ = 0.051), whereas for the G allele carriers, there were no significant associations. The partial regression plots with control for sex are presented in Fig. 5.

To sum up, these results demonstrate that *OXTR* rs53576 polymorphism is associated with emotional aggressive manifestations irrespective of individual’s population background. G allele carriers of the *OXTR* were more predisposed to express anger and hostility than individuals with AA genotype, and this tendency was especially pronounced in men (Fig. 3). At the same time, low prenatal androgen levels (high 2D:4D ratio) also led to higher levels of anger and hostility in both sexes. This result fully corresponds to the sex differences in BPAQ subscales’ ratings, which demonstrate that high levels of anger and hostility are female-specific (Fig. 4). However, the *OXTR* effects on aggression even overweighed those of the prenatal androgens exposure (2D:4D) effects. We came to this conclusion, since the 2D:4D effects were evident only in those individuals who did not have a genetic predisposition to high levels of anger (AA carriers) (Fig. 5).

## Discussion

In this study, we present the data on the role of cultural (ethnic-group), sex, and genetic (*OXTR* rs53576 genotypes) factors in human aggression. According to our knowledge, this is the first study confirming the role of the *OXTR* rs53576 in aggression on the individual level in adult representatives from industrial and traditional ethnic groups.

Our findings supported the hypothesis that (1) self-ratings on aggression are significantly influenced by cultural norms and prescriptions. We demonstrate that ratings on direct (physical and verbal aggression) as well as emotional (anger) and cognitive (hostility) aggression scales were highly culture-specific. To this extent, culture is a “cluster of cognitions, emotions, and practices” [115]. Gender differences in the BPAQ ratings were registered both for industrial and traditional groups. The effect of sex was in the expected direction for physical aggression [13, 15, 17, 116–121]. In Russians, Tatars, Ob-Ugric, Buryats, and Hadza, men rated higher on physical aggression. However, in other traditional African cultures, Datoga and Isanzu, the gender differences in physical aggression were not significant. These findings are important, given the general consensus that universally, this trait is more male-specific as the outcome of sexual selection and social learning [10, 118, 119, 122–126]. In most ethnic groups, gender differences were not found for anger and hostility, with exceptions of anger in Isanzu (higher in women), as well as anger and hostility in Buryats (higher in women). We suggest that the difference obtained in our study reflect cultural norms and socio-cultural network structure. Particularly, relatively high level of ratings on physical aggression in Datoga women may be attributed to the polygynous marriage system and cultural beliefs about individual self-esteem, along with the absence of taboo for the use of physical aggression in conflicts among women, which is especially evident in non-kin interactions [127]. The prevailance of physical aggression in men in most still be due to gender differences in motivational sphere. Men are more motivated to revenge physically, and contra to women, such aggression may be mainly proactive, but not reactive (emotional) [125].

Our data demonstrated that groups of European, African, and Asian origin differ in the distribution of *OXTR* rs53576 genotypes. Buryats and Ob-Ugric, being the most distanced from the rest of our sample, demonstrated a significantly higher prevalence of the AA genotype compared to European and African samples, which is generally in line with previous findings [97]. These differences may suggest certain specific benefits for carriers of the *OXTR* AA genotype in populations with Asian origin as opposed to Europeans and Africans. Earlier, Kim and colleagues hypothesized that the culturally normative behavior might be subjected to genetic influence [128, 129]. According to their findings, the emotional support seeking in distress was typical for the G allele carriers North Americans, compared to AA genotype carriers, but this was not the case for Koreans [32]. At the same time, Koreans having the GG genotype were more likely to use emotional suppression compared to the AA genotype carriers [34]. Supposedly, the G carriers may be more environmentally sensitive. The question then remained whether positive selection in the direction of the AA genotype in Asian populations makes these populations generally more resistant to social-environmental pressure.

Because of cultural-specific differences in social norms and morality, obviously visible in ratings on BPAQ, we conducted the standardization procedure separately for each study sample and used these z-transformed data in further analysis. Given the general sex differences obtained, the effect of the *OXTR* gene polymorphism was tested separately for men and women.

Our findings fully support the hypotheses that (2) the *OXTR* rs53576 polymorphism may cause differences in BPAQ ratings, especially in emotional dimension of aggressive manifestations, and that (3) such effects are sex-specific. We demonstrated that the *OXTR* rs53576 was a significant predictor for ratings on anger and hostility scales, which was more evident for men. The GG carriers rated higher on anger (emotional aggression) and hostility (cognitive aggression) than the AA carriers. Mind that those differences were obtained after minimizing the effect of culture, by z-transformation procedure; hence, higher anger and hostility ratings reveal genetic-associated patterns, rather than cultural-specific differences. Our findings may be of special interest in light of the results, reported on the relationships between the *OXTR* rs53576 polymorphism and general sociality and empathetic abilities, with GG carriers being more empathetic [39, 130]. The association between sensitivity to stressful events with the rs53576 genotypes may be gender-specific. Windle and Mrug reported that young adult female (but not males) carriers of the GG genotype, who previously experienced psychological trauma in adolescence due to parental divorce, had significantly more depressive symptoms, compared to females with AA and AG genotypes [131]. The GG *OXTR* rs53576 genotype carriers were slower in the recognition of emotion of fear compared to the GA genotype [132]. Our findings that GG carriers rated higher on anger and hostility (this tendency was more expressed in men) extend the previous conclusion about higher threshold on emotional sensitivity and lower stress resistance of this *OXTR* rs53576 genotype. Assuming GG genotype carriers are less competent in recognizing of fear in others, they may be more emotionally aggressive (anger) and more hostile to others.

According to our knowledge, this is the first study reporting the interaction effect of oxytocin and prenatal androgenization (assessed through 2D:4D ratio) on the BPAQ anger and hostility ratings. The significant positive effects of the right-hand 2D:4D on anger and hostility were found only for AA carriers and were more evident for men than women. Earlier, Weisman with co-authors suggested that women are doing better in Baron-Cohen’s “Reading the Mind in the Eyes” test (RMET), compared to men due to sex differences in testosterone and oxytocin levels [83]. However, in the study, the main differences were obtained for GG carriers. Particularly, men with higher 2D:4D ratio (low fetal testosterone), performed better on the RMET, possibly because they were better at understanding the emotional state of others than individuals with lower 2D:4D [83]. Our current findings do not support the hypothesis that (4) the presence of the A allele of the *OXTR* plays a crucial role in determining behavioral predispositions, which in turn would distinguish the GG genotype against other variants. A number of studies by other authors have previously reported that AA homozygotes have weaker functional connectivity of the hypothalamus and increased right amygdala activation [130, 133], which most likely affect pro-social behavior and perception. This may suggest that the effects of the A and G alleles could have more or less uniform gradients, with the AA and GG genotypes representing more or less equivalent functional polarities. The physiological processes related to the functioning of the *OXTR* rs53576 locus still need to be investigated in the future.

It is important to consider these results in light of some limitations. The currently accumulated data are inconsistent, obviously demanding replication in other samples with higher power, keeping in mind the small effect sizes, usually reported. Along with testing the gene-environment interaction factor, the gene-gene interaction effect should be considered; hence, the genome-wide association studies may provide much more profound information on the genetic effect on behavior. Currently, the functionality of the *OXTR* rs53576 polymorphism is not clear, as it is located in an untranslated region [134]. At the moment, only associative evidence for the role of *OXTR* in the regulation of brain activity exists. Despite the fact that neuroimaging research has revealed specific brain differences associated with rs53576, such as reduced volumes in hypothalamic gray matter and amygdala for A allele carriers [130, 133, 135], the particular functional role of this very locus remained to be proved in the future studies.

## Conclusions

The ethnic group factor was the most significant predictor of ratings on all four BPAQ scales. All four scales of the BPAQ demonstrated association with sex (main effects), with men scoring higher on physical and verbal aggression and women scoring higher on anger and hostility. After leveling the effects of culture (z-transformation), all four scales of the BPAQ demonstrated association with sex, with men being higher on physical and verbal aggression and women scoring higher on anger and hostility. Anger and hostility scales were also associated with *OXTR* polymorphism and 2D:4D of the right hand. The lowest levels of anger and hostility were observed in individuals with the AA genotype; this effect was especially obvious in men. Both oxytocin (*OXTR* rs53576 polymorphism) and fetal testosterone (2D:4D) significantly affected emotional (anger) and cognitive (hostility) aggression in humans, given the leveling the role of culture.

## Data Availability

The datasets generated and/or analyzed during the current study are available in the Figshare repository, by doi:10.6084/m9.figshare.12442475

## Abbreviations

OXTR: Oxytocin receptor
SNP: Single nucleotide polymorphism
OXTR rs53576: Oxytocin receptor gene single nucleotide polymorphism rs53576
2D:4D: 2nd-to-4th digit ratio
R2D:4D: 2nd-to-4th digit ratio of the right hand
BPAQ: Buss-Perry Aggression Questionnaire
DNA: Deoxyribonucleic acid
GLM: General linear models
RAS: Russian Academy of Sciences
COSTECH: Commission for Science and Technology of Tanzania
HWE: Hardy-Weinberg equilibrium
FST: Fixation index
ANOVA: Analysis of variance
MANOVA: Multivariate analysis of variance
ANCOVA: Analysis of covariance
RMET: Reading the Mind in the Eyes

## References

[1] Boyd R, Richerson PJ (1988). Culture and the evolutionary process.

[2] Carter CS, Cushing BS. Proximate mechanisms regulating sociality and social monogamy, in the context of evolution. In: Chapman AR, Sussman RW, editors. The origins and nature of sociality. New York: Aldine de Gruyter; 2017. p. 99–121.

[3] House BR (2018). How do social norms influence prosocial development?. Curr Opin Psychol..

[4] Fehr E, Gächter S (2002). Altruistic punishment in humans. Nature..

[5] Boyd R (2017). A different kind of animal: how culture transformed our species.

[6] Stanford M. The cultural evolution of human nature. Acta Biotheor. 2019;68(2):1–11. 10.1007/s10441-019-09367-7 PMC7188694 31563992

[7] Chudek M, Henrich J (2011). Culture–gene coevolution, norm-psychology and the emergence of human prosociality. Trends Cogn Sci..

[8] Laland KN, Odling-Smee J, Myles S (2010). How culture shaped the human genome: bringing genetics and the human sciences together. Nat Rev Genet..

[9] Tompson SH, Huff ST, Yoon C, King A, Liberzon I, Kitayama S (2018). The dopamine D4 receptor gene (DRD4) modulates cultural variation in emotional experience. Culture and Brain..

[10] Buss AH, Perry M (1992). The aggression questionnaire. J Pers Soc Psychol..

[11] Farrington DP (1989). Early predictors of adolescent aggression and adult violence. Violence vict..

[12] Valois RF, MacDonald JM, Bretous L, Fischer MA, Drane JW (2002). Risk factors and behaviors associated with adolescent violence and aggression. Am J Health Behav..

[13] Archer J (2004). Sex differences in aggression in real-world settings: a meta-analytic review. Rev Gen Psychol..

[14] Björkqvist K, Österman K, Lagerspetz KM (1994). Sex differences in covert aggression among adults. Aggress Behav..

[15] Butovskaya M, Fedenok J, Burkova V, Manning J (2013). Sex differences in 2D: 4D and aggression in children and adolescents from five regions of Russia. Am J Phys Anthropol..

[16] Butovskaya M, Burkova V, Karelin D, Fink B (2015). Digit ratio (2D: 4D), aggression, and dominance in the Hadza and the Datoga of Tanzania. Am JHum Biol..

[17] Butovskaya M, Burkova V, Karelin D, Filatova V (2019). The association between 2D: 4D ratio and aggression in children and adolescents: cross-cultural and gender differences. Early HumDev..

[18] Heinrichs M, Baumgartner T, Kirschbaum C, Ehlert U (2003). Social support and oxytocin interact to suppress cortisol and subjective responses to psychosocial stress. Biol Psychiatry..

[19] Yang HP, Wang L, Han L, Wang SC (2013). Nonsocial functions of hypothalamic oxytocin. ISRN Neurosci..

[20] Theodoridou A, Rowe AC, Penton-Voak IS, Rogers PJ (2009). Oxytocin and social perception: oxytocin increases perceived facial trustworthiness and attractiveness. Horm Behav..

[21] Lane A, Luminet O, Rimé B, Gross JJ, de Timary P, Mikolajczak M (2013). Oxytocin increases willingness to socially share one’s emotions. Int J Psychol..

[22] Zak PJ, Stanton AA, Ahmadi S (2007). Oxytocin increases generosity in humans. PLOS One..

[23] Shalvi S, De Dreu CK (2014). Oxytocin promotes group-serving dishonesty. PNAS..

[24] Kimura T, Tanizawa O, Mori K, Brownstein MJ, Okayama H (1992). Structure and expression of a human oxytocin receptor. Nature..

[25] Vaidyanathan R, Hammock EAD (2016). Oxytocin receptor dynamics in the brain across development and species. Dev Neurobiol..

[26] Inoue T, Kimura T, Azuma C, Inazawa J, Takemura M, Kikuchi T, Kubota Y, Ogita K, Saji F (1994). Structural organization of the human oxytocin receptor gene. J Biol Chem..

[27] Fragkaki I, Cima M, Verhagen M, Maciejewski DF, Boks MP, Van Lier PA, Koot HM, Branje SJT, Meeus WHJ (2019). Oxytocin receptor gene (OXTR) and deviant peer affiliation: a gene–environment interaction in adolescent antisocial behavior. J Youth Adolesc..

[28] Johansson A, Westberg L, Sandnabba K, Jern P, Salo B, Santtila P (2012). Associations between oxytocin receptor gene (OXTR) polymorphisms and self-reported aggressive behavior and anger: interactions with alcohol consumption. Psychoneuroendocrinology..

[29] Parris MS, Grunebaum MF, Galfalvy HC, Andronikashvili A, Burke AK, Yin H, Min E, Huang Y (2018). Mann JJ Attempted suicide and oxytocin-related gene polymorphisms. J Affect Disord..

[30] Shao D, Zhang HH, Long ZT, Li J, Bai HY, Li JJ, Cao FL (2018). Effect of the interaction between oxytocin receptor gene polymorphism (rs53576) and stressful life events on aggression in Chinese Han adolescents. Psychoneuroendocrinology..

[31] Zhang Y, Wu C, Chang H, Yan Q, Wu L, Yuan S, Xiang J, Hao W, Yu Y (2018). Genetic variants in oxytocin receptor gene (OXTR) and childhood physical abuse collaborate to modify the risk of aggression in Chinese adolescents. J Affect Disord..

[32] Kim HS, Sherman DK, Sasaki JY, Xu J, Chu TQ, Ryu C, Suh EM, Graham K, Taylor SE (2010). Culture, distress, and oxytocin receptor polymorphism (OXTR) interact to influence emotional support seeking. PNAS..

[33] Kim HS, Sherman DK, Taylor SE, Sasaki JY, Chu TQ, Ryu C, Suh EM, Xu J (2010). Culture, serotonin receptor polymorphism and locus of attention. Soc Cogn AffectNeurosci..

[34] Kim HS, Sherman DK, Mojaverian T, Sasaki JY, Park J, Suh EM, Taylor SE (2011). Gene-culture interaction: oxytocin receptor polymorphism (OXTR) and emotion regulation. Soc Psychol Pers Sci..

[35] Kim HS, Sasaki JY (2014). Cultural neuroscience: biology of the mind in cultural context. Ann Rev Psychol..

[36] Sasaki JY, Kim HS (2017). Nature, nurture, and their interplay: a review of cultural neuroscience. J Cross-Cult Psychol..

[37] Ishii K, Masuda T, Matsunaga M, Noguchi Y, Yamasue H, Ohtsubo Y. Do culture and oxytocin receptor polymorphisms interact to influence emotional expressivity? Cult Brain. 2020. 10.1007/s40167-020-00091-5.

[38] Monin JK, Goktas SO, Kershaw T, DeWan A (2019). Associations between spouses’ oxytocin receptor gene polymorphism, attachment security, and marital satisfaction. PloS One..

[39] Li J, Zhao Y, Li R, Broster LS, Zhou C, Yang S (2015). Association of oxytocin receptor gene (OXTR) rs53576 polymorphism with sociality: a meta-analysis. PloS One..

[40] Conner TS, McFarlane KG, Choukri M, Riordan BC, Flett JAM, Phipps-Green AJ, Topless RK, Merriman ME, Merriman TR (2018). The oxytocin receptor gene (OXTR) variant rs53576 is not related to emotional traits or states in young adults. Front. Psychol..

[41] Berenbaum SA, Korman Bryk K, Nowak N, Quigley CA, Moffat S (2009). Fingers as a marker of prenatal androgen exposure. Endocrinology..

[42] Malas MA, Dogan S, Evcil EH, Desdicioglu K (2006). Fetal development of the hand, digits and digit ratio (2D: 4D). Early Hum Dev..

[43] McIntyre MH (2006). The use of digit ratios as markers for perinatal androgen action. Reprod Biol Endocrin..

[44] Gooding DC, Chambers BH (2018). Age of pubertal onset and 2nd to 4th digit ratios: preliminary findings. Early Hum Dev..

[45] Lombardo MP, Thorpe PA, Brown BM, Sian K (2008). Digit ratio in birds. Anat Rec..

[46] Direnzo GV, Stynoski JL (2012). Patterns of second-to-fourth digit length ratios (2D: 4D) in two species of frogs and two species of lizards at La Selva, Costa Rica. Anat Rec.

[47] Abbott AD, Colman RJ, Tiefenthaler R, Dumesic DA, Abbott DH (2012). Early-to-mid gestation fetal testosterone increases right hand 2D∶ 4D finger length ratio in polycystic ovary syndrome-like monkeys. PloS one..

[48] Baxter A, Wood EK, Jarman P, Cameron AN, Capitanio JP, Higley JD (2018). Sex differences in rhesus monkeys’ digit ratio (2D: 4D ratio) and its association with maternal social dominance rank. Front Behav Neurosci..

[49] Manning JT, Barley L, Walton J, Lewis-Jones DI, Trivers RL, Singh D, Thornhill R, Rohde P, Bereszkei T, Henzi P, Soler M, Szwed A (2000). The 2nd:4th digit ratio, sexual dimorphism, population differences, and reproductive success. Evol Hum Behav..

[50] Hönekopp J, Watson S (2010). Meta-analysis of digit ratio 2D:4D shows greater sex difference in the right hand. Am J Hum Biol..

[51] Manning JT, Fink B (2020). Understanding COVID-19: Digit ratio (2D: 4D) and sex differences in national case fatality rates. Early Hum Dev..

[52] Kratochvíl L, Flegr J (2009). Differences in the 2nd to 4th digit length ratio in humans reflect shifts along the common allometric line. Biol. Lett..

[53] Lolli L, Batterham AM, Kratochvíl L, Flegr J, Weston KL, Atkinson G (2017). A comprehensive allometric analysis of 2nd digit length to 4th digit length in humans. P. Roy. Soc. B-Biol. Sci..

[54] Forstmeier W. Avoiding misinterpretation of regression lines in allometry: is sexual dimorphism in digit ratio spurious? BioRxiv. 2018;298786.

[55] Lutchmaya S, Baron-Cohen S, Raggatt P, Knickmeyer R, Manning JT (2004). 2nd to 4th digit ratios, fetal testosterone and estradiol. Early Hum Dev..

[56] Ventura T, Gomes MC, Pita A, Neto MT, Taylor A (2013). Digit ratio (2D:4D) in newborns: influences of prenatal testosterone and maternal environment. Early Hum Dev.

[57] Richards G (2017). What is the evidence for a link between digit ratio (2D:4D) and direct measures of prenatal sex hormones?. Early Hum Dev.

[58] Zheng Z, Cohn MJ (2011). Developmental basis of sexually dimorphic digit ratios. PNAS..

[59] Medland SE, Zayats T, Glaser B, Nyholt DR, Gordon SD, Wright MJ, Montgomery GW, Campbell MJ, Henders AK, Timpson NJ, Peltonen L, Wolke D, Ring SM, Deloukas P, Martin NG, Davey Smith J (2010). Evans DM A variant in LIN28B is associated with 2D: 4D finger-length ratio, a putative retrospective biomarker of prenatal testosterone exposure. Am J Hum Genet..

[60] Lawrance-Owen AJ, Bargary G, Bosten JM, Goodbourn PT, Hogg RE, Mollon JD (2013). Genetic association suggests that SMOC1 mediates between prenatal sex hormones and digit ratio. Hum Genet..

[61] Zhanbing M, Jie D, Chunyue B, Hong L, Liang P, Zhenghao H (2019). Association of CYP19A1 single-nucleotide polymorphism with digit ratio (2D: 4D) in a sample of men and women from Ningxia (China). Early Hum Dev..

[62] Codesal J, Regadera J, Nistal M, Regadera-Sejas J, Paniagua R (1990). Involution of human fetal Leydig cells. Animmunohistochemical, ultrastructural and quantitative study. J Anat..

[63] Dong L, Jelinsky SA, Finger JN, Johnston DS, Kopf GS, Sottas CM, Hardy MP, GE RS. (2007). Gene expression during development of fetal and adult Leydig cells. Ann NY Acad Sci..

[64] Wu X, Wan S, Lee MM (2007). Key factors in the regulation of fetal and postnatal Leydig cell development. J Cell Physiol..

[65] Hönekopp J, Bartholdt L, Beier L, Liebert A (2007). Second to fourth digit length ratio (2D:4D) and adult sex hormone levels: new data and a meta-analytic review. Psychoneuroendocrinology.

[66] Zhang K, Yang X, Zhang M, Wang C, Fang P, Xue M, Zhao J, Gao X, Pan R, Gong P (2020). Revisiting the relationships of 2D: 4D with androgen receptor (AR) gene and current testosterone levels: replication study and meta-analyses. J Neurosci Res..

[67] Kowal M, Sorokowski P, Żelaźniewicz A, Nowak J, Orzechowski S, Żurek G, Żurek A, Juszkiewicz A, Wojtycka L, Sieniuć W, Poniatowska M, Tarnowska K, Kowalska K, Drabik K, Łukaszek P, Krawczyk K, Stefaniak T, Danek N (2020). No relationship between the digit ratios (2D: 4D) and salivary testosterone change: study on men under an acute exercise. Sci Rep..

[68] Chi JG, Dooling EC, Gilles FH (1977). Left-right asymmetries of the temporal speech areas of the human fetus. Arch Neurol..

[69] Dubb A, Gur R, Avants B, Gee J (2003). Characterization of sexual dimorphism in the human corpus callosum. Neuroimage..

[70] Cosgrove KP, Mazure CM, Staley JK (2007). Evolving knowledge of sex differences in brain structure, function, and chemistry. Biological Psychiatry..

[71] Zuloaga DG, Puts DA, Jordan CL, Breedlove SM (2008). The role of androgen receptors in the masculinization of brain and: what we’ve learned from the testicular feminization mutation. Horm Behav..

[72] Salmon CA, Hehman JA (2018). Second to fourth digit ratio (2D: 4D), tomboyism, and temperament. Pers Individ Differ..

[73] Bailey AA, Hurd PL (2005). Finger length ratio (2D: 4D) correlates with physical aggression in men but not in women. BiolPsychol..

[74] Butovskaya ML, Fry DP (2013). Aggression and conflict resolution among the nomadic Hadza of Tanzania as compared with their pastoralist neighbors. War, peace, and human nature: the convergence of evolutionary and cultural views.

[75] Butovskaya ML, Burkova VN, Rostovtseva VV, Fedenok YN, Gonsales TM, de la Cruz AR, Butovskaya PR, Lazebny OE (2019). Aggression self-assessment indicators and its association with sex, confession and FKBP5 rs1360780 polymorphism. Voprosy Psikhologii.

[76] Turanovic JJ, Pratt TC, Piquero AR (2017). Exposure to fetal testosterone, aggression, and violent behavior: a meta-analysis of the 2D: 4D digit ratio. Aggress Violent Behav..

[77] Hoskin AW, Meldrum RC (2018). The association between fetal testosterone and violent behavior: additional evidence using the 2D: 4D digit ratio. Pers Individ Differ..

[78] Butovskaya ML, Apalkova YI, Fedenok JN (2017). 2D:4D, self-rated aggression, risk taking and personality traits in parachutists. Vestn. Mosk. Univ., Ser. 23. Anthropol.

[79] Kozieł S, Kociuba M, Chakraborty R, Sitek A, Ignasiak Z (2018). Further evidence of an association between low second-to-fourth digit ratio (2d: 4d) and selection for the uniformed services: a study among police personnel in Wrocław, Poland. J Biosoc Sci.

[80] Brañas-Garza P, Galizzi MM, Nieboer J (2018). Experimental and self-reported measures of risk taking and digit ratio (2d: 4d): evidence from a large, systematic study. Int Econom Rev..

[81] Barel E (2019). 2D: 4D, optimism, and risk taking. Curr Psychol..

[82] Hönekopp J, Watson S (2011). Meta-analysis of the relationship between digit-ratio 2D: 4D and aggression. Pers IndividDiffer..

[83] Weisman O, Pelphrey KA, Leckman JF, Feldman R, Lu Y, Chong A, Chen Y, Monakhov M, Hong Chew S, Ebstein RP (2015). The association between 2D: 4D ratio and cognitive empathy is contingent on a common polymorphism in the oxytocin receptor gene (OXTR rs53576). Psychoneuroendocrinology..

[84] Kret ME, De Dreu CKW (2013). Oxytocin-motivated ally selection is moderated by fetal testosterone exposure and empathic concern. Front. Neurosci..

[85] Gossen A, Hahn A, Westphal L, Prinz S, Schultz RT, Gründer G, Spreckelmeyer KN (2012). Oxytocin plasma concentrations after single intranasal oxytocin administration–a study in healthy men. Neuropeptides..

[86] Rhee SH, Waldma ID, Shaver PR, Mikulincer M (2011). Genetic and environmental influences on aggression. Herzilya series on personality and social psychology. Human aggression and violence: Causes, manifestations, and consequences. American Psychological Association.

[87] Butovskaya ML, Vasilyev VA, Lazebny OE, Burkova VN, Kulikov AM, Mabulla A, Shibalev DV, Ryskov AP (2012). Aggression, digit ratio, and variation in the androgen receptor, serotonin transporter, and dopamine D4 receptor genes in African foragers: the Hadza. Behav Genet..

[88] Butovskaya ML, Lazebny OE, Vasilyev VA, Dronova DA, Karelin DV, Mabulla AZ, Shibalev DV, Shackelford TK, Fink B, Ryskov AP (2015). Androgen receptor gene polymorphism, aggression, and reproduction in Tanzanian foragers and pastoralists. PloS One..

[89] Butovskaya ML, Butovskaya PR, Vasilyev VA, Sukhodolskaya JM, Fekhredtinova DI, Karelin DV, Fedenok JN, Mabulla AZP, Ryskov AP, Lazebny OE (2018). Serotonergic gene polymorphisms (5-HTTLPR, 5 HTR1A, 5 HTR2A), and population differences in aggression: traditional (Hadza and Datoga) and industrial (Russians) populations compared. J Physiol Anthropol..

[90] van Donkelaar M, Bralten J, Buitelaar J, Hoogman M, Franke B (2019). ENIGMA2 Consortium, EAGLE Consortium. Neural mechanisms mediating gene-behavior associations in aggression. Eur Neuropsychopharm..

[91] Parlapani E, Nasika Z, Kyriazis O, Nimatoudis I, Fountoulakis KN, Nimatoudis I (2019). Genetics and behaviour. Psychobiology of Behaviour.

[92] Zhang-James Y, Fernàndez-Castillo N, Hess JL, Malki K, Glatt SJ, Cormand B, Faraone SV (2019). An integrated analysis of genes and functional pathways for aggression in human and rodent models. Mol Psychiatry..

[93] Ruisch IH, Dietrich A, Klein M, Faraone SV, Oosterlaan J, Buitelaar JK, Hoekstra PJ (2020). Aggression based genome-wide, glutamatergic, dopaminergic and neuroendocrine polygenic risk scores predict callous-unemotional traits. Neuropsychopharmacology..

[94] Poore HE, Waldman ID (2020). The association of oxytocin receptor gene (OXTR) polymorphisms antisocial behavior: a meta-analysis. Behav Genet..

[95] Sasaki JY, Kim HS, Xu J (2011). Religion and well-being: the moderating role of culture and the oxytocin receptor (OXTR) gene. J Cross-Cult Psychol..

[96] Rostovtseva V, Butovskaya M, Mkrtchjan R (2019). 2d: 4d, Big fives and aggression in young men of Caucasian, Ural and Asian origin. Soc Evol Hist..

[97] Butovskaya PR, Lazebny OE, Sukhodolskaya EM, Vasiliev VA, Dronova DA, Fedenok JN, Rosa A, Peletskaya EN, Ryskov AP, Butovskaya ML (2016). Polymorphisms of two loci at the oxytocin receptor gene in populations of Africa, Asia and South Europe. BMC Genet.

[98] Rostovtseva VV, Mezentseva AA, Windhager S, Butovskaya ML. Sexual dimorphism in facial shape of modern Buryats of Southern Siberia. Am J Hum Biol. 2020:e23458. 10.1002/ajhb.23458. 10.1002/ajhb.23458 32596969

[99] Sukhodolskaya EM, Fehretdinova DI, Shibalev DV, Lazebny OE, Mabulla AZ, Butovskaya ML, Ryskov AP, Vasilyev VV (2018). Polymorphisms of dopamine receptor genes DRD2 and DRD4 in African populations of Hadza and Datoga differing in the level of culturally permitted aggression. Ann Hum Genet..

[100] Sukhodolskaya EM, Vasilyev VA, Shibalev DV, Shcherbakova OI, Kulikov AM, Lazebny OE, Karelin DV, Butovskaya ML, Ryskov AP (2015). Comparative analysis of polymorphisms of the serotonin receptor genes HTR1A, HTR2A, and HTR1B in Hadza and Datoga males. Russ J Genet..

[101] Vasilyev VA, Sukhodolskaya EM, Kulidzhanov PV, Kulikov AM, Lazebny OE, Dronova DA, Butovskaya ML, Shibalev DV, Ryskov AP (2014). Polymorphism of 5-HTTLPR and Stin2 loci of the serotonin transporter gene in males of African ethnic populations Hadza and Datoga. Russ J Genet..

[102] Fekhretdinova DI, Sukhodolskaya EM, Shibalev DV, Lazebnyy OE, Butovskaya ML, Ryskov AP, Vasil’yev VA (2018). Polymorphism of the two genes encoding catecholamine degradation enzymes (COMT and MAOA) in the Hadza and Datoga African ethnic populations. Mol Gen Microbiol Virol..

[103] Butovskaya ML, Vasilyev VA, Lazebny OE, Suchodolskaya EM, Shibalev DV, Kulikov AM, Karelin DV, Burkova VN, Mabulla A, Ryskov AP (2013). Aggression and polymorphisms in AR, DAT1, DRD2, and COMT genes in Datoga pastoralists of Tanzania. Sci Rep..

[104] Butovskaya M, Sorokowska A, Karwowski M, Sabiniewicz A, Fedenok J, Dronova D, Negasheva M, Selivanova E, Sorokowski P (2017). Waist-to-hip ratio, body-mass index, age and number of children in seven traditional societies. Sci Rep..

[105] Butovskaya M, Marczak M, Misiak M, Karelin D, Białek M, Sorokowski P (2020). Approach to resource management and physical strength predict differences in helping: evidence from two small-scale societies. Front Psychol..

[106] Peakall R, Smouse PE (2012). GenAlEx 6.5: genetic analysis in Excel. Population genetic software for teaching and research-an update. Bioinformatics..

[107] Peakall R, Smouse PE (2006). GENALEX 6: genetic analysis in Excel. Population genetic software for teaching and research. Mol Ecol Notes..

[108] Brislin RW (1970). Back-translation for cross-cultural research. J Cross-Cult Psychol..

[109] Chapman DW, Carter JF (1979). Translation procedures for the cross cultural use of measurement instruments. Educ Eval Policy Anal..

[110] Manning JT, Fink B (2008). Digit ratio (2D: 4D), dominance, reproductive success, asymmetry, and sociosexuality in the BBC Internet Study. Am J Hum Biol..

[111] Fink B, Manning JT, Neave N, Tan U (2004). Second to fourth digit ratio and hand skill in Austrian children. Biol. Psychol..

[112] Manning JT, Scutt D, Wilson J, Lewis-Jones DI (1998). The ratio of 2nd to 4th digit length: a predictor of sperm numbers and concentrations of testosterone, luteinizing hormone and oestrogen. Human Reprod..

[113] Shingala MC, Rajyaguru A (2015). Comparison of post hoc tests for unequal variance. Int J Innov Res Sci Eng Technol..

[114] Dunnett CW (1980). Pairwise multiple comparisons in the unequal variance case. J Am Stat Assoc..

[115] Korotaev AV (2004). World religions and social evolution of the old world Oikumene civilizations: a cross-cultural perspective.

[116] Archer J (2009). Does sexual selection explain human sex differences in aggression?. Behav Brain Sci..

[117] Liu J, Zhou Y, Wenyu GU. Reliability and validity of Chinese version of Buss-Perry aggression questionnaire in adolescents. Chin J Clin Psychol. 2000;4.

[118] Gerevich J, Bácskai E, Czobor P (2007). The generalizability of the Buss–Perry aggression questionnaire. Int J Meth Psych Res..

[119] Morales-Vives F, Vigil-Colet A (2010). Are there sex differences in physical aggression in the elderly?. Pers Individ Differ..

[120] Reyna C, Sanchez A, Ivacevich MGL, Brussino S (2011). The Buss-Perry Aggression Questionnaire: construct validity and gender invariance among Argentinean adolescents. Int J Psychol Res..

[121] Butovskaya ML, Timentschik VM, Burkova VN (2007). Aggression, conflict resolution, popularity, and attitude to school in Russian adolescents. Aggress Behav..

[122] Bernstein IH, Gesn PR (1997). On the dimensionality of the Buss/Perry aggression questionnaire. Behav Res Ther..

[123] Harris MB, Knight-Bohnhoff K (1996). Gender and aggression II: personal aggressiveness. Sex Roles..

[124] Ramirez JM, Andreu JM, Fujihara T (2001). Cultural and sex differences in aggression: a comparison between Japanese and Spanish students using two different inventories. Aggress Behav..

[125] Wilkowski BM, Hartung CM, Crowe SE, Chai CA (2012). Men don’t just get mad; they get even: revenge but not anger mediates gender differences in physical aggression. J Res Pers..

[126] Madran PHAD (2013). The reliability and validity of the Buss-Perry Aggression Questionnaire (BAQ)-Turkish version. Turk Psikiyatri Dergisi..

[127] Butovskaya ML (2012). Wife battering and traditional methods of its control in contemporary Datoga pastoralists of Tanzania. J Aggress Confl Peace Res..

[128] Kim HS, Sherman DK, Taylor SE (2008). Culture and social support. Am Psychol..

[129] Taylor SE, Sherman DK, Kim HS, Jarcho J, Takagi K, Dunagan MS (2004). Culture and social support: who seeks it and why?. J Pers Soc Psychol..

[130] Tost H, Kolachana B, Hakimi S, Lemaitre H, Verchinski BA, Mattay VS, Weinberger DR, Meyer–Lindenberg A (2010). A common allele in the oxytocin receptor gene (OXTR) impacts prosocial temperament and human hypothalamic-limbic structure and function. PNAS..

[131] Windle M, Mrug S (2015). Hypothesis-driven research for G× E interactions: the relationship between oxytocin, parental divorce during adolescence, and depression in young adulthood. Front Psychol..

[132] Stanković M, Bašić J, Milošević V, Nešić M (2019). Oxytocin receptor (OXTR) gene polymorphisms and recognition memory for emotional and neutral faces: a pilot study. Learn Motiv..

[133] Wang J, Qin W, Liu B, Wang D, Zhang Y, Jiang T, Yu C (2013). Variant in OXTR gene and functional connectivity of the hypothalamus in normal subjects. Neuroimage..

[134] Tops S, Habel U, Radke S (2019). Genetic and epigenetic regulatory mechanisms of the oxytocin receptor gene (OXTR) and the (clinical) implications for social behavior. Horm Behav..

[135] Wang J, Qin W, Liu B, Zhou Y, Wang D, Zhang Y, Jiang Y, Yu C (2014). Neural mechanisms of oxytocin receptor gene mediating anxiety-related temperament. Brain Struct Funct..

